# Preadolescents’ healthy eating behavior: peeping through the social norms approach

**DOI:** 10.1186/s12889-020-09366-1

**Published:** 2020-08-20

**Authors:** Tija Ragelienė, Alice Grønhøj

**Affiliations:** Department of Management, Aarhus University, School of Business and Social Sciences, Aarhus, Denmark

**Keywords:** Peers relationships, Social norms, Self-efficacy, Preadolescents, Healthy eating

## Abstract

**Background:**

The rising childhood obesity rate is a major public health challenge. The objective of this study is to examine key underlying mechanisms for peer-related social influence on preadolescents’ healthy eating behavior by including factors closely linked with the quality of preadolescents’ relationship with peers.

**Methods:**

A cross-sectional study was conducted in a convenience sample of 278 Lithuanian preadolescents, recruited from a public school. A questionnaire containing sociodemographic questions, questions about food intake, peer-related social norms of healthy eating, social self-efficacy, vegetable preference, need for peer approval and feeling of belonging were applied. Data was analyzed using structural equation modeling.

**Results:**

The results of the SEM showed that social self-efficacy predicts feeling of belonging to the peer group and need for peer approval. Feeling of belonging and need for peer approval predict actual intake of vegetables via injunctive norms of healthy eating. However, neither feeling of belonging nor need for peer approval predicted descriptive norms of healthy eating. Contrary to our expectations, descriptive norms were found to be unrelated with actual intake of vegetables, though vegetable preference predicted actual intake of vegetables. Vegetable preference was not predicted by injunctive or descriptive peers’ social norms of healthy eating.

**Conclusions:**

The findings of this study offer insight for informing parents, teachers and for social norms marketing interventions by stressing the importance of social relations when the aim is to encourage healthy eating among preadolescents.

## Background

Many children and adolescents do not consume the recommended amounts of fruit and vegetables [1–3], and this, in turn, contributes to the increasing incidence of childhood obesity worldwide [4, 5]. According to the World Health Organization [6], over 340 million children and adolescents aged 5–19 were overweight or obese in 2016 corresponding to a rate of 1 in 5 children and adolescents. Childhood obesity may result in detrimental health conditions in adult age and is very likely to be transmitted into adulthood [7, 8]. Being overweight or obese in childhood is linked with many serious non-communicable diseases such as diabetes, hypertension or cardiovascular diseases [9]. Therefore, it is crucial to deepen our knowledge of the causes of overweight and obesity to prevent civilization diseases [10] and to cultivate a healthier and happier society.

In early adolescence when parental influence lessens, many adolescents start to develop eating habits that are influenced by their peer group [11], and the social influence of peers is considered to be important for shaping children’s and preadolescents’ food preferences and eating behavior [12–15]. It has been found that adolescents and their best friends exhibit similarities in healthy eating patterns [16], and peers’ approval and attitudes of food choice are significant predictors of eating behavior [17]. Peer encouragement to eat healthily has been linked with higher frequencies of consumption of healthy foods and lower frequencies of consumption of unhealthy food items in adolescence [18]. Children’s and adolescents’ taste preferences, food choices, and actual eating behavior can also be impacted by peers’ injunctive [19–22] and descriptive [23–25] norms. Following peers’ social norms of eating might be related with modelling peers’ behavior since social norms can serve as environmental cues that regulate the intake of food [26], especially in those cases where individuals want to ally with a peer model, or perceive themselves to be similar to the model [27, 28]. Children have been found to adjust their food intake under the influence of peers to model social eating behavior [29], and qualitative studies have indicated that adolescents consume food in order to fit in with peers [30] or due to peer pressure [31]. Also, adolescents have been reported to use food choices to create a desired image and to indicate their compliance with common friendship and peer norms [31].

On the other hand, other studies have found that peers did not influence children’s dietary behavior [32, 33] or adolescents’ fruit and vegetable intake significantly [34]. Similarly, adolescents’ positive perceptions of healthily-eating peers were not a significant predictor of their eating behavior [35]. No relationship was found between healthy food intake and friends’ encouragement to eat high-fat foods or sweets, or praising high-fat foods or sweets, and friends’ social support for healthy eating and healthy food intake [36]. Though most research has shown a significant peer influence on eating among children and adolescents, the evidence is mixed regarding the nature of this influence, and further theoretical and empirical work is needed in this area [37]. Similarly, though social influence has been recognized to play a role for the prevalence of obesity [38], little is known of how such social influence could be conceptualized, and the mechanisms through which social factors can influence eating behavior are still unclear [39]. Therefore, the psychosocial basis of peers’ influence on children’s and adolescents’ eating behavior still needs further exploration.

Social norms theories posit that behavior is influenced by perceptions of how members of our social group think and act [18, 40]. *Injunctive* social norms indicate a perception of what other group members would consider as appropriate behavior. *Descriptive* social norms refer to the perception of what most group members do [40]. Normative social influence denotes the mechanism by which individuals alter their behavior and attitudes in order to comply with group norms [27, 28]. Social pressure, social norms, and hierarchies that have an impact on adolescents’ social experiences, arise mostly from the peer group itself [41]. Social norms emerge from attributes that are significant for the group [42] and define what is normative in the group and foster compliance and homogeneity among group members [43]. The transmission of norms through group affiliation combines the subjective and structural dimensions of connectedness. The subjective dimension of connectedness (or in other words, feeling of belonging to the group) may impact how adolescents recognize value on norms since a large body of research has suggested that adolescents’ norms and behaviors are greatly affected by friends and by those to whom they strive to be closely associated [44]. The socialization process or group impact can either be apparent as it is the case of peer pressure and actual support or disapproval, or subtle and implicit acting via group norms, expectations, social acceptance and status associated with particular behaviors [42]. Adolescents tend to adjust their behavior towards the group norm showing general susceptibility to peer influence [45].

The importance of social norms for shaping individual behavior is related with the concept of self-categorization and perception of psychological group membership [39]. Belonging to a group gives people a feeling of social connectedness as the outcome of a psychological process where individuals gradually perceive themselves as being a part of the group and therefore start to define their selves in terms of their social identities [46]. This could explain the developing preadolescent’s need to belong to a peer group and [47] get their approval [48]. Thus, a feeling of peer group belonging assumes a central role for sustaining well-being in the developing social world of adolescents, who are especially sensitive to social exclusion in this unique period of life [49]. The need to belong to a group can affect an adolescent’s behavior before he or she becomes an actual member of the group because individuals tend to change their behavior in order to gain peer approval. Therefore, peer group affiliation does not necessarily need to be reciprocal in order to influence one’s behavior [49]. The need for peer approval can be defined as a person’s tendency to accept or reject social pressure [50]. Adolescents’ need for peer approval may potentially influence the quality of their relationships with peers in a negative way, though at the same time peer approval is extremely important for adolescents at a period of time when attention to the opinion of peers intensifies [51].

The concepts of belongingness to the peer group and need for peer approval can help describe the quality of preadolescents’ relationships with peers (in the opposite direction as above). Preadolescents’ social self-efficacy is vital in order to establish and maintain satisfying and sound relationships with peers [52]. According to Bandura [53], social self-efficacy beliefs include the awareness of proper social behaviors, confidence in one’s ability to engage in the behaviors adequately, and faith that the responses to one’s efforts to interact will attract the necessary support from one’s social environment. Therefore, preadolescents’ social self-efficacy may be part of the mechanism that can explain peers’ social influence through the characteristics of the quality of relationship with peers, thereby shaping one’s behavior according to the social norms of the relevant peer group. This mechanism may also be used to explain preadolescents’ eating behavior and their food choices. Social self-efficacy could be linked to factors indicating the quality of the relationship with peers (for example, the need for peer approval and belonging to the peer group) which in turn can explain the conformity with peers’ social norms. This could contribute to explaining social influence on preadolescent’s eating behavior.

**The objective** of this study is to examine the mechanisms that could explain peers’ social influence on preadolescents’ healthy eating behavior (actual intake of vegetables and vegetable preferences) in the context of children’s relationships with peers. Peer-related social norms of healthy eating and factors that are closely linked with the quality of preadolescents’ relationship with peers are hypothesized to predict preadolescent’s healthy eating behavior.

## Methods

### Participants

Elementary and secondary school children from the 3rd to the 6th grades in the administrative regions of Kaunas, Lithuania, participated in this study during December, 2018; they were recruited from conveniently selected public school. All participants were Lithuanian and spoke Lithuanian at home. The project was registered at Aarhus University and is in accordance with internal ethical guidelines and complies with the principles embodied in the Helsinki declaration. Written parental consent and informed consent from participants were obtained before conducting the research.

### Procedure

The selected school was visited before the study took place in order to arrange the time and date of assessment with the school administration and inform participants about it. During the introductory meeting, children were informed that participation is voluntary, and the research aim was presented in simple language appropriate for the target age group. Letters were used to inform parents about the study. Parents were asked to return the signed consent form to the school and written consent to participate was provided from parents or guardians of all participants. After obtaining the informed consent of children’s parents and children themselves, self-reported questionnaires were administered by the researcher at the school during regular class hours. It took from 30 to 40 min to complete the questionnaire. Each participant that completed a questionnaire received a small gift for participating.

### Measures

The variables of the conceptual model were assessed using instruments validated in previous research with children or adolescents, or using direct questions. Sociodemographic questions about participants’ age (years), sex (boys, girls), parents’ education (university degree, no university degree), working status (employed, unemployed) and family composition (both parents, mother only, father only, guardians) were also included in the questionnaire. All scales used in this study were freely available from published papers with no special permissions required for their use.

### Perceived social norms of healthy eating in the peer group

Perceived descriptive and injunctive social norms were measured using 5-point Likert type items, one item for each type of norms. The item used for descriptive norms was “*Most of the kids I know in my school eat healthy food like fruit and vegetables”.* The item used for injunctive norms was *“Most of my friends think that I should eat healthily*.” The responses varied from “Strongly disagree” to “Strongly agree” (adapted from [54]).

### Vegetable intake

Daily intake of fruit, vegetables and snacks was measured with 5-point Likert type, one item each. However, only measures for vegetables were included in the subsequent analyses, since items needed to test the hypotheses of the present study were only included for vegetables and not for fruit and snacks. For the vegetable intake measure, the item used was “*How many serving spoons of cooked or raw vegetables do you eat on an average day?”* The responses varied from “None or less than one serving per day” to “more than four servings per day”.

### Vegetable preference

To evaluate preadolescents’ attitudes towards healthy eating, a vegetable preference subscale from food preference questionnaire was used [55]. Participants were asked to rate their liking of 16 individual foods (vegetables) on a 5-point Likert scale, ranging from “not at all” to “a lot”. Participants were instructed to select ‘not applicable’ if they were not familiar with, or had no memory of having tried a food item. Cronbach’s alpha for vegetable preference in this sample was .85.

### Social self-efficacy

This was evaluated using a subscale of social self-efficacy from a self-efficacy questionnaire (*SEQ-C*) for children [56]. The social self-efficacy subscale contains eight items that are hypothesized to represent the domain of social self-efficacy (perceived capability for peer relationships and assertiveness). Scores were measured on a 5-point Likert scale with 1 = *not at all* and 5 = *very well.* (e.g., *“How easy is it for you to become friends with other children?”*). Cronbach alpha for the subscale was .76.

### Feeling of belonging to the peer group

This was assessed using a peer group-integration subscale from the Relational Provision Loneliness Questionnaire (RPLQ) [57]. Seven items were used to assess participants’ feeling of belonging in relation to peers. Participants were asked to indicate to what extent each of seven statements (e.g., *“I am outgoing and friendly with other kids in my class.”*) was true for them ([57], p. 46) on a 5-point Likert scale scores ranged from *not at all* to *very true of me*. Cronbach alpha for this subscale was .89.

### Need for peer approval

We applied 10 items of the need for peer approval scale [50]. Participants were asked to choose if the statements were “true” or “false” as regarded them (e.g. *“I hang out with my friends if they ask me, even when I have a lot of homework to do.”*), where “true” was coded as 1, and “false” was coded as 0. Since one item-total correlation was negative, it was excluded from the scale and in total nine items were used for further analysis. Cronbach’s alpha in this sample was .54, which suggests that need for peer approval is not equally consistent in this sample, but it is still reasonable to assume that the scale is reliable and suitable for group comparisons. According to Smith, Kendall and Hulin [58] and Hinton, Brownlow, McMurray & Cozens [59], Cronbach’s alpha above .50 indicates a satisfactory reliability of the scale which is legitimate and acceptable with a short scale (less than 20 items) [60].

### Conceptual model

In our conceptual model, higher self-efficacy is hypothesized to be associated with stronger feeling of belonging to the peer group and a less need to seek peer approval, since social self-efficacy has been linked with better interpersonal relationships with peers [61] and with better communication and interpersonal problem-solving skills [62]. Considering preadolescents’ susceptibility for peer approval and the increasing concern for peers’ opinions [51] during adolescence, their desire to belong to a peer group and to gain peer acceptance [49], it is expected that feeling of belonging to peers and need for peer approval predict perceived descriptive and injunctive peers’ social norms of healthy eating. Given that children and adolescents’ food choices, taste preferences and actual eating behavior can be influenced by descriptive [23–25] and injunctive peer social norms [19–22], and that adolescents tend to adapt their behavior to fit with peer group norms [45], we assumed that descriptive and injunctive social norms are related with preadolescents’ actual intake of vegetables and vegetable preferences, which, in turn, also predict the actual intake of vegetables.

### Data analysis

To describe sample population characteristics, calculations of mean and percentage were applied. To compare the mean scores of healthy eating behavior variables between sex (boys vs. girls), and different age groups (younger vs. older participants) we used Student t test. Participants were divided into younger and older groups according to their mean age. Pearson correlation test was used to examine relations between study variables. Analyses were performed using SPSS software (IBM SPSS Statistics, version 20.0). Results were considered to be significant at the *p* < 0.05 level.

Structural equation modelling (SEM) was used to estimate the fit of the survey data to the conceptual model (the data can be obtained from the corresponding author). Before starting data analysis, data were checked for normality and outliers, since normality of distribution is one of the assumptions for the maximum likelihood method to be used in SEM analysis [63]. The distribution of study variables corresponded to the normality assumptions [64] and could be regarded as adequately normal for further analyses. Since missing values appeared completely at random, they were all left blank. The missing data of different variables ranged from 0 to 2.9%. Harman’s [65] single-factor test was performed to evaluate the extent of the common method variance in the dataset [66]. All survey items were entered into a principal component factor analysis with no rotation and factor and a fixed number of one factor was extracted. Unrotated factor solution analysis showed that one emerging factor explained only 17.04% of the variance, which implies that common method variance is unproblematic in this study. The second test of common method variance was performed using a procedure described by Eichhorn [67]. The results of the analysis indicated that the common method variance was not a concern in our study [66, 67].

The SEM analysis was carried out in two steps, the first involving an assessment of the suitability of the measurement model and the second fitting the structural equation model. Indicators made from item parcels of the latent factors were used for the identifying measurement model because parcels can generate more reliable latent variables than individual items [68]. Using the random assignment technique recommended by Little, Rhemtulla, Gibson, and Schoemann [69], parcels were designed in identical ways across each scale of latent variables. The measurement model and hypothesized conceptual model were specified and estimated using R package “*Lavaan”* in version 3.5.1 “*Feather Spray*”.

## Results

### Sample

Parents’ consent rate to let their child participate in the survey was 98.02%, participants’ consent (considering only those children whose parent signed the consent) rate was 96, and 2.68% of participants whose parents signed the consent forms were not present during the survey. The sociodemographic characteristics of participants are presented in Table 1. A total of 278 (46% boys) participants filled in the questionnaire. Respondents’ age ranged between 8 and 13 years of age (M = 10.61, SD = 1.11). Most children (80%) reported living in full families with both parents, and that both

**Table 1:** *Sociodemographic characteristics of study participants (N = 278)*

Characteristics	Mean (SD) or N (%)
**Boys, n**	129 (46)
**Age, years**	10.61 (1.11)
**Preadolescent lives with, n**	
Both parents	222 (80)
Mother only	52 (19)
Father only	2 (less than 1)
Guardians	1 (less than 1)
No answer	1 (less than 1)
**Parents working status, n**	
Both parents are working	219 (79)
Mother only	32 (12)
Father only	24 (9)
Both parents are unemployed	2 (less than 1)
No answer	1 (less than 1)
**Mother’s education, n**	
University degree	221 (80)
No university degree	31 (11)
No answer	26 (9)
**Father’s education, n**	
University degree	191 (69)
No university degree	48 (17)
No answer	39 (14)

Mean, standard deviation (SD), percentage

parents were currently employed (79%).

### Comparison of study variables for gender and age groups

No difference was observed between boys and girls with regard to actual intake of fruit, vegetables, vegetable preferences, social self-efficacy, need for peer approval, feeling of belonging to the peer group, and descriptive and injunctive norms. Therefore, it was decided not to distinguish groups by gender in further structural equation analysis. No differences were observed in different age groups of injunctive and descriptive norms and social self-efficacy. In the group of younger participants, the means of injunctive and descriptive norms were significantly higher compared to the older participants group. The mean of social self-efficacy was higher in the older participants group (see Table 2).

**Table 2:** *Comparison of mean scores of preadolescent’s relationships with peers and healthy eating behavior variables for younger and older participants groups (N = 278)*

	Younger participants (*N* = 118)		Older participants (*N* = 160)		
	Mean	SD	Mean	SD	*p* value
Social self-efficacy	3.49	0.67	3.68	0.65	**.02**
Need for peer approval	0.57	0.21	0.52	0.25	.08
Feeling of belonging to the peers’ group	3.67	0.93	3.85	0.77	.12
Descriptive norms of healthy eating	3.43	0.86	2.99	0.89	**.0001**
Injunctive norms of healthy eating	3.34	1.17	2.79	1.15	**.0001**
Vegetable preferences	2.95	0.92	2.90	0.91	.702
Actual intake of vegetables	1.77	1.36	1.73	1.29	.801

Student t test, scales used by [56, 50, 57, 54, 55];

### Correlations between preadolescents’ peer relationships and healthy eating behavior variables

Social self-efficacy was positively related with feeling of belonging to peers and negatively with the need for peer approval. Social self-efficacy was also positively correlated with vegetable preferences and actual vegetable intake. The need for peer approval was negatively correlated with feeling of belonging to peers and descriptive norms. Feeling of belonging to peers correlated positively with descriptive norms of healthy eating and actual vegetable intake. Injunctive norms were positively related with the need for peer approval and actual vegetable intake (see Table 3).

**Table 3:** *Correlations between preadolescents’ relationship with peers and healthy eating behavior variables for overall sample (N = 278)*

	1.	2.	3.	4.	5.	6.	7.
1. Social self-efficacy	1.00						
2. Need for peer approval	−.441**	1.00					
3. Feeling of belonging to the peers’ group	.708**	−.439**	1.00				
4. Descriptive norms of healthy eating	.151*	−.125*	.192**	1.00			
5. Injunctive norms of healthy eating	−.037	.132*	.069	.389**	1.00		
6. Vegetable preferences	.254**	−.062	.199**	.088	.073	1.00	
7. Actual intake of vegetables	.232**	−.044	.228**	.063	.192**	.208**	1.00

Pearson correlation test coefficients, *p < .05, **p < .01, ***p **< .001.** Scales used by [56] [50]; [57] [54]; ;[55];

Concerning sociodemographic factors, living with both parents was significantly related with actual vegetable intake (*r* = 0.158, *p* < 0.01). For this reason, this variable was tested for confounding effects. Living with both parents was not statistically significantly associated with the exposure variable (social self-efficacy) in the model (*r* = .071, *p* = .238) and was therefore not involved as a covariate to control for the potential confounding effect while testing the structural equation model. None of the other sociodemographic variables (parents’ education, preadolescents’ age, parents’ working status) were statistically significantly related with preadolescents’ vegetable preference, actual vegetable intake and were therefore also excluded as covariates in the testing of the structural equation model.

### Model testing

The fit indices of the confirmatory factor analysis of the measurement model showed a good fit of the data to the model. Chi-square (df = 48) =63.92, *p* = .062, RMSEA = .035 (90% confidence interval (CI): .000–.056), CFI = .98, and SRMR = .039. All factor coefficients of the measurement model were statistically significant at *p* < .0001. The full measurement model with standardized factor loadings are presented in Fig. 2 in the Appendix.

**Fig. 1:**
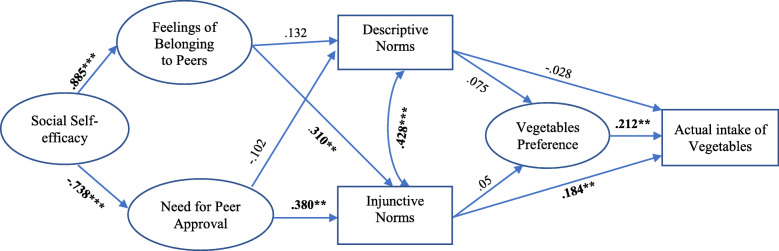
Structural equation model of the relationships between hypothesized factors and vegetables intake patterns (standardized estimates) ***p < 0.001, **p < 0.01, *p < 0.05

**Fig. 2:**
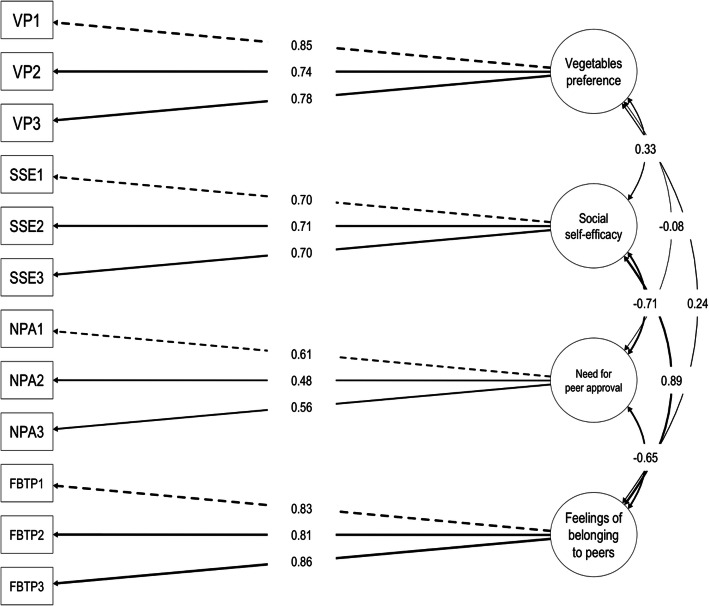
Confirmatory Factor Analysis of a Measurement Model of Study Variables. Standardized Parameters

**Fig. 3:**
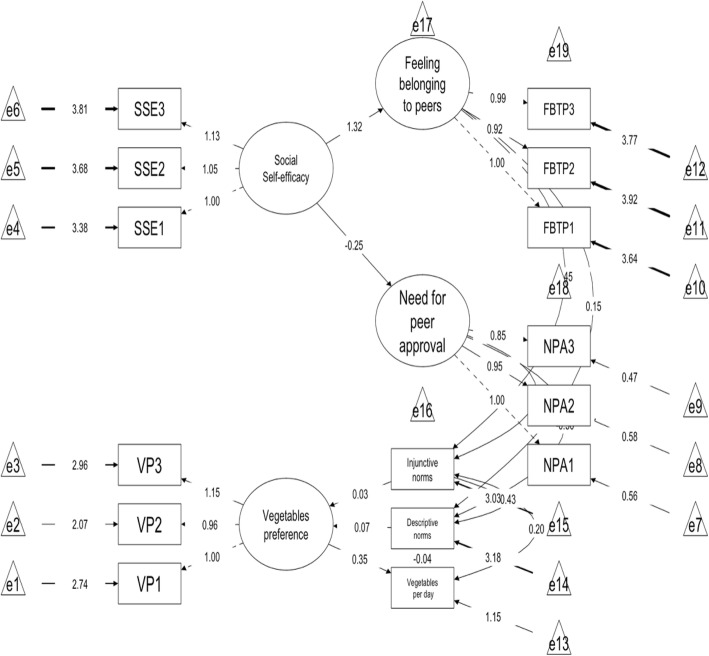
Structural Equation Model of Pre-adolescents Healthy Eating Behavior. Unstandardized Parameters

The graphical model of structural model is presented in Fig. 1 (full structural model equation model is presented in Fig. 3 in the Appendix). In the hypothesized model, social self-efficacy, feeling of belonging to peers, need for peer approval, descriptive and injunctive perceived social norms for healthy eating, and vegetable preferences were tested as latent constructs in the structural model to control for measurement error, and the actual intake of vegetables was tested as directly observed variable. The structural equation model of healthy eating behavior showed a good fit to the data, Chi^2^ (df = 81) = 124.01, *p* = .001, RMSEA = .044 (90% confidence interval (CI): .027–.059), CFI = .97, and SRMR = .08.

The path coefficients from social self-efficacy to feeling of belonging to peers (ß = .885, *p* < .001) and need for peer approval (ß = -.738, *p* < .001) were significant. However, the feeling of belonging to peers (ß = .132, *p* > .05) and the need for peer approval were not significantly related with descriptive norms of healthy eating (ß = -.102, *p* > .05). The feeling of belonging to peers (ß = .310, *p* < .01) and the need for peer approval (ß = .380, *p* < .05) positively predicted injunctive social norms of healthy eating. It was also discovered that paths from injunctive norms of healthy eating to actual vegetable intake (ß = .184, *p* < .05); and from vegetable preference to actual vegetable intake (ß = .212, *p* < 0.01) were statistically significant. However, paths from descriptive norms of healthy eating to vegetable preference (ß = .075, *p* > .05) and from descriptive norms to actual vegetable intake (ß = -.028, *p* > .05), as well as the path from injunctive social norms to vegetable preferences were not significant (ß = -.05, *p* > .05).

## Discussion

The findings of this study partly support the conceptualized model of explaining preadolescents’ healthy eating behavior through the relationships with peers and perceived eating norms of peers. Social self-efficacy was strongly related with preadolescents’ need for peer approval in a negative direction, but positively related with feeling of belonging to a peer group. As expected, feeling of belonging to peers and need for peer approval were directly related to injunctive norms of healthy eating, which in turn were related to the actual intake of vegetables. Contrary to expectations, feeling of belonging to peers and need for peer approval were not related to descriptive norms of healthy eating, and descriptive norms did not predict vegetable preferences nor actual intake of vegetables. Neither injunctive nor descriptive norms of healthy eating were predictors of vegetable preference; however, vegetable preference was a direct and positive predictor of preadolescents’ actual vegetable intake.

The positive correlation between social self-efficacy and preadolescents’ feeling of belonging to the peer group could be related to the fact that adolescents with a higher level of social self-efficacy are capable of maintaining better interpersonal relationships with peers [61], and also their communication and interpersonal problem-solving skills tend to be better [62]. This is also in line with Bandura’s [70] social cognitive theory and previous studies that have emphasized the importance of social self-efficacy in maintaining good peer relationships [52, 61, 62, 71, 72]. The negative path of social self-efficacy and need for peer approval could be explained by low social self-efficacy and the increased likelihood of adolescents being involved in peer victimization [71], peer neglect and withdrawal from peers, anxious and sad feelings of neglect [72], may make preadolescents more susceptible to peer pressure and fulfilling of peer expectations [73, 74].

Significant paths from feeling of belonging to peers and need for peer approval to injunctive norms in the model could be related to the nature of perception of injunctive norms. Injunctive norms that take place in a social context and which are external to the subject, not only display behaviors that others approve but also that compliance with these norms are encouraged and violations from them will be punished [75, 76], for example by exclusion from the peer group for not complying with the social norms of the group [77, 78]. Acceptance, approval, and belongingness to the peer group are essential for preadolescents’ successful psychosocial development [49, 79, 80], especially considering the fact that at the age of 9–13 years, preadolescents are extremely susceptible to being rejected by peers [81]. Another explanation could be related to preadolescents’ self-categorization and perception of psychological membership to the peer group, since perception of shared identity with the communicators of social norms can determine the conformity with the social norms of eating, while the social context determines which norms and identities are likely to be salient [39, 82].

In line with previous studies [19, 21, 22], injunctive norms were related with the actual intake of vegetables. This may be related to that fact that injunctive norm messages likely affect behavior by making prominent already existing beliefs [83], and eating a lot of fruit and vegetables can be one of preadolescents’ internalized beliefs, which may be taught in school when learning about healthy diet, or by parents at home. Another explanation could be that injunctive norms are thought to be advantageous because they help people’s goal of affiliation. Through strategic action, such as complying with injunctive norms, people aim to gain social approval and avoid disapproval and other negative social sanctions (Cialdini & Goldstein, 2004; Cialdini et al., 1990 as cited in [84]), and this fits well with preadolescents’ need for peer approval and belongingness to the peer group.

No significant paths from feeling of belonging to peers and need for peer approval to descriptive norms were indicated in this study. This could be related to the fact that in this particular period of life4, preadolescents are very susceptible to peer pressure as regards their health behavior and decision-making [85–89], and therefore, injunctive peer norms may surpass the influence of descriptive norms since preadolescents try to fit in and be accepted by the peer group. Also, descriptive norms may be used by adolescents as an indicator of injunctive norms, since most people (including children and adolescents) approve of eating fruit and vegetables even if they do not eat many vegetables [90]. Additionally, descriptive and injunctive norms are related to each other and with the actual eating behavior [54]. A high, significant correlation between descriptive and injunctive norms of healthy eating was also found in this study.

In contrast to the results of previous research [23, 91–94], no significant paths were found from perceived descriptive norms of healthy eating to vegetable preference and actual intake of vegetables. This may be explained by injunctive norms of healthy eating being more important for explaining preadolescents’ actual vegetable intake in the context of social relationships with peers. Despite preadolescents’ believing that peers think eating fruit and vegetables is a good thing, they were actually not modeling this behavior or encouraging the eating of fruit and vegetables in real-life situations [95]. Also, though social models can influence eating behavior through the descriptive social norms, people may be unaware of this influence on their food intake [96]. Another explanation might be that the social and symbolic meanings which adolescents relate with healthy eating, may differ with processes and values such as fitting in with the peer group or self-image [31]. This means that adolescents’ wish to be accepted and belong to the peer group have a huge influence on their food choices, but this impact may occur more implicitly, considering that belongingness to peers and getting their approval may be more important for adolescents than food consumption itself.

Contrary to expectations and findings of previous research [23, 24, 97, 98], the results of this study revealed that neither injunctive nor descriptive norms of healthy eating predicted vegetable preference. This may be related to the fact that food preferences tend to remain stable throughout childhood [99]. Food preferences established in childhood [100] have been shown to continue into adulthood [101], which may explain why perceived social norms of healthy eating did not predict preadolescents’ vegetable preference significantly, because food preferences are formed as a result of early childhood experiences with food, positive and negative conditioning, food exposure, and genetic predispositions to different tastes [102]. It could also be that generally children tend to believe that food preferences and eating norms are aligned. Consequently, in order to comply with peer norms, it is possible that children make choices that do not show their own preferences [103]. Some studies also indicate only weak-to-moderate correlations between children and adolescents and their friends’ food preferences, since low resemblance of food preferences was found in peer dyads [104]. The results of this study showed that vegetable preference is a significant predictor of preadolescents’ actual vegetable intake. This is in line with other studies indicating that children’s eating patterns are strongly associated with their food preferences [105, 106] and is a strong determinant of children and adolescents’ healthy eating [107]. This may be associated with an inborn component of food preferences, as children seem to have a preference for sweet foods and a dislike for foods that taste bitter (for example, vegetables) [108, 109].

Though peer influence on preadolescent eating behavior is discussed in the scientific literature, the mechanism of peers’ impact on children and adolescents eating behavior is still not fully determined. Theories of social norms highlight the significance of descriptive and injunctive norms and constitute the most widely used approaches in explaining peer influence. Many previous studies have confirmed that descriptive and injunctive social norms have an impact on children’s and adolescents’ eating behavior and food choice [19, 21, 22, 110–113]. This study contributes to the field by giving a better understanding of preadolescents’ healthy eating behavior in the context of social relationship with peers highlighting the importance of preadolescents’ need for belongingness and peer group approval, and explaining mechanism of how social norms can influence preadolescents’ vegetable intake.

Our findings suggest that interventions that address preadolescents’ social self-efficacy and aspects of relationships with peers, such as injunctive norms for making healthy food choices, may be beneficial for preadolescents since social influence could potentially be used for enhancing healthy eating behavior [82]. Interventions that aim to encourage preadolescent healthy eating behavior could appeal to create new and healthier social norms and aim to create a favorable environment where preadolescents can foster sound social relationships with their peers so that one of their most important needs – to have good relationships with peers and being accepted by them – would be met. Interventions should also help preadolescents develop social communication and interpersonal problem-solving skills in order to increase their level of social self-efficacy and use the peer context to promote healthy eating behavior with implying new and “healthy” social norms into preadolescent groups. In this sense, healthy eating interventions using a social relationship approach would not only be applicable for adolescents with health problems but also for the general population of adolescents, for instance when pending sustainable and healthy dietary guidelines are to be implemented [114]. Finally, the wider ramifications of cultivating social self-efficacy and “healthy” relationships with peers should be made clear for parents and teachers.

As this study was cross-sectional, it is not possible to claim causality of the tested relations between variables. Another limitation is the generalizability of our findings. The sample was formed of preadolescents who varied in age from 8 to 13 years living in the second-largest city in Lithuania. In this study, the conceptual model was only tested in one sample of preadolescents without separating children into age or grade specific groups. It is worth mentioning that in the model tested, only two aspects that describe the quality of preadolescent relationship with peers (need for peer approval and belongingness to peer group) were explored. Another potential limitation is that in the model tested only perceived peer norms of healthy eating were included; future studies could include parents’, siblings’ or even teachers’ social norms as control variables to explain peers’ impact on preadolescents’ healthy eating behavior. Additionally, the Cronbach’s alpha of need for peer approval scale was relatively low in this study and the quality of the measures of factors describing quality of preadolescents’ relationship with peers need improvement for future studies. In this study, only vegetable preferences and vegetable intake were used as healthy eating behavior indicators. This study relied only on preadolescents’ self-report, and this might be another limitation.

Collecting data about preadolescents’ eating habits from social agents close to the preadolescents’ environment, such as parents, friends, and teachers, may extend the understanding of the social norm mechanism of preadolescents’ healthy eating behavior. Future research could include other important relationship factors such as peer pressure, susceptibility to peer influence or identity with the particular peer group, which were not examined in this study. Future studies could also test the model of explaining peers’ impact on children’s healthy eating behavior using other indicators that are usually described as related with healthy eating, for example, intake of and preferences for fruit and dairy products [106, 115, 116]. It could also be interesting to see if the model could explain preadolescents’ snacks and calorie-dense food choices and preferences.

## Conclusion

Social self-efficacy was positively associated with preadolescents’ belongingness to peer groups, whereas it was negatively associated with the need for peer group approval. Belongingness to the peer group and need for peer approval were positively linked with preadolescents perceived injunctive peer norms of healthy eating, which in turn were associated with higher vegetable intake. Neither belongingness to peers nor need for peer approval were significantly associated with perceived descriptive norms of peers’ healthy eating. Descriptive norms were not related to preadolescents’ intake of vegetables. Neither injunctive nor descriptive social norms predicted preadolescents’ vegetable preferences. However, preadolescents’ vegetable preferences were a significant predictor of the actual intake of vegetables. The findings of this study can be used for informing parents, teachers and for social norms marketing interventions by stressing the importance of social relations when the aim is to encourage healthy eating among preadolescents.

## Data Availability

The data used in this study is available on Zenodo repository: https://zenodo.org/record/3973197

## Abbreviations

SEM: Structural equation modelling
CI: Confidence interval
CFI: Comparative fit index
RMSEA: Root mean square error of approximation
SRMR: Standardized Root Mean Square Residual
